# Redefining exposure science to advance research supporting cumulative impacts, environmental justice, and decision-making

**DOI:** 10.1038/s41370-023-00610-5

**Published:** 2023-11-09

**Authors:** Nicolle S. Tulve, Annette Guiseppi-Elie, Andrew M. Geller, Cavin K. Ward-Caviness, Sean J. Paul, Emma T. Lavoie, Louie Rivers, H. Christopher Frey

**Affiliations:** 1 U.S. Environmental Protection Agency, Office of Research and Development, Research Triangle Park, NC USA; 2 U.S. Environmental Protection Agency, Office of Research and Development, Washington, DC USA

**Fig. 1:**
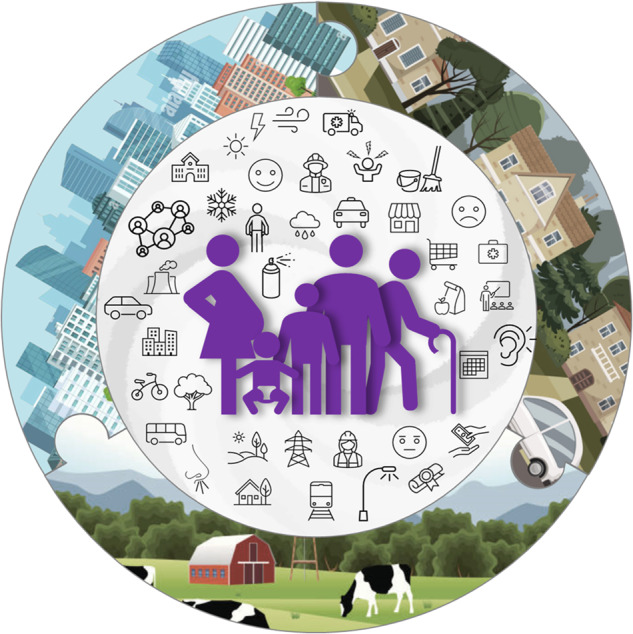
Pictorial depiction of the definition of exposure science. Exposure to both chemical and non-chemical stressors may occur at any lifestage throughout the lifecourse in every community. Icons representing both chemical and non-chemical stressors are shown swirling around the lifecourse figure. The border represents any urban, suburban, or rural community.

Exposure science is often described as characterizing and predicting the intersection of chemical, biological, and physical agents with receptors, including individuals, geographically defined groups, and communities, in both space and time [1]. However, to address today’s complex scientific challenges, it is imperative that the working definition of exposure science be holistic, consistent with the concept of the exposome [2], and inclusive of the importance of non-chemical stressors (Fig. 1). Thus, the definition should characterize and predict the intersection of agents, including chemical and non-chemical stressors, with receptors in both space and time. By expanding the definition of exposure science to include non-chemical stressors, the scientific community stresses the importance of understanding how all exposures relate to health, well-being, and quality of life outcomes.

Extreme weather events resulting from climate change are increasingly common and impact all communities by threatening the essential ingredients for good health, well-being, and quality of life, such as clean air, safe drinking water, and clean land. Climate change exacerbates the inequitable distribution of chemical and other non-chemical stressors that result in disproportionate exposures and greater vulnerability to already overburdened communities. Stressors include not only chemical contaminants in air, water, and land (e.g., particulates in air, perfluorinated compounds in water, metals in soil), but also non-chemical stressors (e.g., climatic stressors, relationships and social connectivity, access to resources such as health care and food stores, public infrastructure, job stability) associated with the built, natural, and social environments.

Overburdened communities often contain historically marginalized groups and include “fenceline communities” that are adjacent to facilities that discharge chemicals into the environment [3–9]. Reports published by the U.S. EPA [3], World Health Organization [10], and American Lung Association [11], amongst others, describe efforts to identify and characterize cumulative impacts that affect overburdened communities. Cumulative impacts describes the concept that health, well-being, and quality of life outcomes are dependent on a wide variety of factors that can occur concurrently and persistently in a person’s life. Without a collective scientifically-based effort to identify, characterize, and mitigate exposures experienced by those most disproportionately impacted, these communities will continue to experience worse health, well-being, and quality of life outcomes when compared to communities not considered overburdened. Scientific research must employ systems approaches for studies of environmental health that support understanding and eliminating health disparities. Thus, we must study interrelated aspects of the built, natural, and social environments that affect health, well-being, and quality of life outcomes as strongly as we study the links between exposures to chemicals and health.

The challenge to the exposure science community can be summarized in three overarching questions: (1) How do we work to improve human health and the environment when we have the complex array of stressors that define the exposome to consider?; (2) What is the correct balance between foundational research, methodological development, and translational science to identify, characterize, and mitigate the most important stressors and improve community health and well-being?; and (3) How can incorporation of the exposome into exposure science guide decisions that lead to actionable solutions to protect the most vulnerable communities?

Exposure scientists play a critical role in informing action toward community resilience and advancing environmental justice. The multiple, disproportionate, and cumulative impacts experienced by overburdened communities need to be addressed collectively through multidisciplinary efforts led by scientists in government, academia, industry, and non-governmental organizations. Community-based participatory science contributes to the development of qualitative and quantitative place-based information needed to inform exposure characterization [12]. A whole-of-science approach is needed to inform decisions to reduce the stressors experienced by overburdened communities. The scientific community has the opportunity to make connections between individual research efforts and this broader research agenda.

In 2022, there were many examples of extreme weather events (e.g., the floods in Pakistan, heat waves in England, droughts in China, heavy rainfall resulting in mudslides in South Africa, and wildfires in the western United States), adversely impacting already overburdened communities around the world. Combined exposures to multiple stressors, exacerbated by climate change, result in both direct and indirect health effects such as: changes in disease burden; rendering land uninhabitable; reducing crop yield; heat stress on human health; overwhelming drinking water and storm water infrastructure; and increasing the likelihood of exposures to chemicals and air pollutants [4, 13–17]. Extreme weather events exacerbate many factors, such as economic and social distress, resulting in disproportionate impacts to the health, well-being, and quality of life in already overburdened communities.

President Biden signed four Executive Orders (EOs) (13985 [18], 14008 [19], 14091 [20], and 14096 [21]) to advance racial equity and support for underserved communities and to address disproportionate impacts and the climate crisis in the United States. These EOs provide a framework for stimulating action across the U.S. Federal government to address health inequities from disproportionate exposures to chemical agents and environmental degradation magnified by racial, economic, and geographic factors, in combination with climate change. EPA Administrator Regan embraced President Biden’s commitment to environmental justice when he stated “At EPA, we believe that every person in the United States has the right to clean air, clean water, and a healthier life—no matter how much money they have in their pocket, the color of their skin, or their zip code” [22].

The U.S. EPA’s mission is to protect human health and the environment. This mission is served by understanding, characterizing, and reducing health risks associated with exposures to chemical and non-chemical stressors [23]. The U.S. EPA works to ensure that all Americans have clean air, land, and water in the everyday environments where they live, learn, play, and work [24]. At the foundation of the U.S. EPA’s mission are four core principles: follow the science, follow the law, be transparent, and advance racial justice and equity [25]. To support the Agency’s mission, the Office of Research and Development (ORD) provides the data, tools, and scientific information that are the foundation for credible decision-making [26]. ORD has prioritized research to advance the scientific community’s understanding of the complex interrelationships between chemical and non-chemical stressors, in support of community health.

ORD exposure scientists have described the interrelationships between chemical and non-chemical stressors of the exposome, and how they affect health, well-being, and quality of life outcomes, as key components of the Total Environment framework. The Total Environment framework is a systems approach to understand the interrelationships between chemical and non-chemical stressors from the built, natural, and social environments, activities and behaviors, and inherent characteristics in affecting health, well-being, and quality of life outcomes [27]. Within the Total Environment framework, there are any number of ways to describe the components that make up the built, natural, and social environments, including but not limited to social, economic, infrastructure, institutional, community, environmental, climatic, and cultural components [28]. Collectively, this totality of exposures and their effects on health, well-being, and quality of life outcomes describe cumulative impacts [29].

Recent ORD contributions to the literature highlight associations between exposures to multiple stressors, living in adverse neighborhood environments, and health and well-being [30–32]. Martin et al. [30] showed that a molecular measure of biological aging, known as epigenetic aging or DNA methylation age, was influenced by aspects of the built and social environments experienced by members of the community. Ward-Caviness et al. showed that air pollution, which disproportionately impacts historically marginalized communities, also accelerates the aging process according to DNA methylation age [31]. Ward-Caviness et al. showed that accelerated aging may be a biomarker of sensitivity to air pollution, such that individuals with accelerated aging have worse responses to traffic-related air pollution [32].

Across these series of manuscripts, researchers demonstrated that exposures to chemicals and negative social experiences (e.g., non-chemical stressors) can be encoded into one’s DNA and accelerate the aging process. Biomarkers of accelerated aging are also indicative of worsened responses to future exposures such as traffic-related air pollution. Thus, cumulative impacts from the social and chemical environments can directly impact health as well as modify how an individual responds to future exposures.

The exposure science community is uniquely suited to advance research relevant to policy considerations at the nexus of chemical and non-chemical stressors, cumulative impacts, environmental justice, and decision-making. Collectively, we can improve the understanding of non-chemical stressors, links between chemical and non-chemical stressors, how stressors modify biological response(s), and provide information on how exposures to stressors might be prevented, mitigated, or reduced. We recognize the complex dynamics around environmental inequities and appreciate that social, economic, environmental, physical, and health sciences and engineering are all integral in identifying solutions to this complex challenge. Exposure science is well poised to help the scientific community focus on solutions to help communities across the globe, inform decisions that protect the most vulnerable members of society, and incorporate the most pressing exposure science needs related to environmental and social injustices and cumulative impacts.
